# Hot cross bun sign in a case with multisystem atrophy

**Published:** 2014-04-03

**Authors:** Mohammad Rohani

**Affiliations:** ^1^ Department of Neurology, School of Medicine, Rasoul Akram Hospital, Iran University of Medical Sciences, Tehran, Iran

**Keywords:** Hot Cross Bun Sign, Magnetic Resonance Imaging, Multiple System Atrophy, Parkinsonism

A 65-year-old man came to our clinic with progressive disequilibrium and gait problem since 6 years. During this period, he had also urinary incontinency.

On neurologic examination, he had scanning speech and bidirectional nystagmus. There was hypokinesia in upper and lower limbs and on cerebellar tests finger to nose and heel to shin were impaired (severe dysmetria); he was unable to walk without bilateral support.

**Figure 1:**
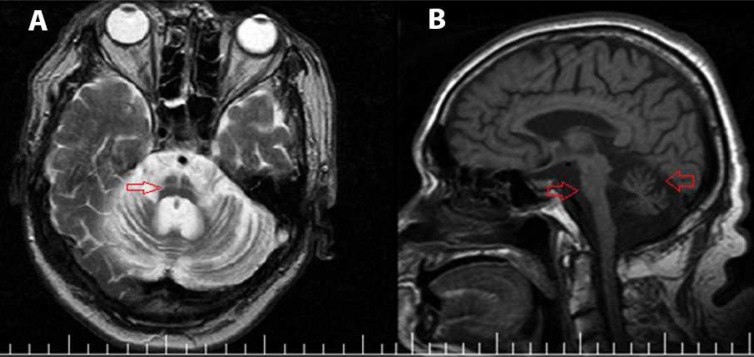
A: Axial T2-weighted imaging shows severe cerebellar atrophy and a typical hot cross bun sign in pons (red arrow). B: Sagittal T1-weighted magnetic resonance imaging shows severe cerebellar and pontine atrophy (red arrows).

The brain magnetic resonance imaging (MRI) of the patient showed severe cerebellar and pontine atrophy and a typical hot cross bun sign in pons, which is typical for MSA (Figure 1).

Characteristic features of MSA on brain MRI are hyperintensity and thinning of putamen, narrowing and hyperintensity of posterolateral part of putamen, atrophy of brainstem (especially pons) and cross hyperintensity of pons (hot cross bun sign). The cause of hot cross bun sign is degeneration of pontine neurons and pontocerebellar fibers.
